# Upper Jaw Mucormycosis in an Immunocompetent Patient: A Clinical Report

**DOI:** 10.1155/crdi/8920048

**Published:** 2026-04-05

**Authors:** Chloé Thepenier, Adélaïde Carlier, Louis Brochet, Emma Bach, Pierre Bouletreau, Andrea Varazzani

**Affiliations:** ^1^ Maxillofacial Surgery Department, Lyon Sud Hospital, Hospices Civils de Lyon, Lyon, France, chu-lyon.fr

**Keywords:** CAM, case report, immunocompetent, mucormycosis, rhino-orbital

## Abstract

Mucormycosis is a rare but severe opportunistic infection, whose incidence has substantially increased since the coronavirus disease 2019 (COVID‐19) pandemic. It predominantly affects immunocompromised patients, individuals with diabetes, and those receiving corticosteroids. Standard treatment involves extensive debridement of affected tissue combined with prolonged antifungal therapy. We present a case of an immunocompetent patient with no identifiable risk factors who developed maxillary mucormycosis associated with COVID‐19 illness. The condition responded favorably to minimal debridement and antifungal treatment. This case raises questions about the need for extensive debridement in immunocompetent patients without underlying risk factors.

## 1. Introduction

Mucormycosis is a rare but serious fungal infection caused by molds of the order *Mucorales*. These molds are found in the environment, particularly in soil, decaying organic matter, and various surfaces, including manufactured products [1]. The infection is typically acquired through inhalation, occasionally through ingestion of contaminated food, or via wounds [2]. Although these molds do not usually threaten immunocompetent individuals, they function as opportunistic pathogens in those with weakened immunity or underlying health conditions. Mucormycosis infections are characterized by rapid progression, which can lead to severe complications and death if not diagnosed and treated promptly. The infection invades soft tissue, nerves, bone, and cartilage, resulting in tissue infarction, vessel thrombosis, and necrosis [3]. The most common forms of mucormycosis include rhino‐orbito‐cerebral, cutaneous, disseminated, gastrointestinal, and pulmonary infections.

Rhino‐orbital mucormycosis typically begins in the nasal sinuses and may extend to the orbits and surrounding structures. Patients present with symptoms such as nasal congestion, facial pain, swelling, and black nasal discharge. Progression can lead to ocular symptoms, including proptosis, loss of vision, and ophthalmoplegia. The associated mortality is substantial, estimated at approximately 20% in patients without systemic disease and up to 80% in those with underlying conditions [4]. Diagnosis relies on a combination of clinical assessment, imaging studies (computed tomography [CT] scans or magnetic resonance imaging [MRI]), and biopsies.

The primary risk factor for mucormycosis infection is immunosuppression. This encompasses patients with uncontrolled diabetes, which contributes to elevated blood glucose levels, creating an environment conducive to fungal proliferation, those with neutropenic hematological disorders, organ transplant recipients, and individuals receiving immunosuppressive medications (including corticosteroids). Other reported risk factors include iron overload, physical trauma (e.g., burns or surgery), broad‐spectrum antibiotic use, and chronic renal failure [5].

Although mucormycosis is predominantly reported in immunocompromised individuals, cases in immunocompetent patients account for an estimated 4–19% [6]. Recent literature specifically analyzing rhino‐cerebral disease in apparently healthy individuals includes 49 such cases, demonstrating an overall survival rate of 91.8%. Intracranial involvement was the only independent predictor of mortality in this cohort [7]. In the pulmonary domain, a 2024 study reviewed 14 cases of pulmonary mucormycosis in immunocompetent hosts and documented high mortality despite aggressive therapy, highlighting that normal immune status does not eliminate the risk of infection [8]. Additional reports describe cutaneous or gastrointestinal mucormycosis following trauma or environmental exposure in patients without overt immunodeficiency.

Several additional reports specifically highlight maxillary, rhino‐orbital, or craniofacial mucormycosis in immunocompetent patients. For example, a recent case report described an unusual presentation of rhino‐orbital‐cutaneous mucormycosis in an immunocompetent patient, who developed extensive cutaneous and orbital disease requiring surgical exenteration and prolonged antifungal therapy [9]. Chronic and destructive rhino‐orbital mucormycosis has also been reported in an otherwise healthy 59‐year‐old patient with progressive vision loss, highlighting that delayed diagnosis can contribute to severe local invasion, even in the absence of systemic risk factors [10]. Similarly, a rare case of rhinomaxillary mucormycosis involving the hard palate in a 45‐year‐old immunocompetent woman, initially misdiagnosed as aspergillosis and requiring aggressive surgical resection and targeted antifungal therapy, was reported [11]. In line with these observations, coronavirus disease 2019 (COVID‐19)–associated mucormycosis (CAM) has been reported in immunocompetent patients as a delayed complication, including cases of maxillary involvement with bony sequestration attributable to fungal‐induced vascular thrombosis and necrosis [12]. Such cases reinforce that systemic immunocompetence does not preclude severe Mucorales infection and that careful documentation of unique presentations is required.

Hypotheses for the development of mucormycosis in immunocompetent hosts include local inoculation through breaches in mucosal or cutaneous barriers with a high inoculum (including dental procedures or surgical wounds) [13], local microenvironmental changes that transiently favor fungal growth, such as increased glucose, acidic pH, and elevated free iron (for example, after tissue ischemia or microhemorrhage), endothelial injuries (including severe viral infections, ischemia, or inflammation), and dysregulated local immunity [14] or unrecognized and subtle immune defects [15]. Finally, recent syntheses favor a multihit model, involving a combination of several low‐grade risk modifiers (for example, transient hyperglycemia after steroid use, a contaminated device delivering a high inoculum, local tissue ischemia, and age‐related immune senescence) [13].

This paper describes a case of mucormycosis affecting the upper jaw in an immunocompetent patient following a mild COVID‐19 infection.

This case has been reported in line with the CARE criteria.

## 2. Case Presentation

### 2.1. Patient Presentation

A 65‐year‐old man was referred to the Maxillofacial Surgery Department of the Lyon Sud Hospital, France, by a stomatologist after a cone beam computed tomography (CBCT) scan revealed a maxillary sequestrum. His medical history included nonmetastatic testicular cancer treated with chemotherapy 4 years prior, hypertension managed with combination therapy, and antiplatelet therapy after iliac stent placement. He denied substance abuse and had no immunosuppressive conditions.

### 2.2. Clinical Examination

The oral mucosa appeared normal; however, vestibular fistulas with intermittent purulence were noted around teeth 15, 12, 21, and 22. Tenderness upon vestibular palpation and infracentimetric jugulo‐carotid lymph nodes were observed bilaterally. The patient was afebrile.

### 2.3. Timeline

The patient reported experiencing left hemifacial pain for 3 months after a mild case of COVID‐19. Initial imaging, including MRI performed to rule out trigeminal autonomic cephalalgia and a CT scan, suggested ethmoido‐maxillary sinusitis without bone involvement.

Recurrent periodontal abscesses during the subsequent month led to multiple courses of antibiotic therapy. No periodontal cause was identified. Block‐like mobility of teeth 25 and 26 resulted in their extraction and the removal of a bone sequestrum. Because infectious symptoms and dental mobility persisted, the patient consulted a stomatologist. A CBCT scan revealed a large maxillary bone sequestrum, prompting referral to our department.

### 2.4. Diagnostic Assessment and Interpretation

**FIGURE 1:**
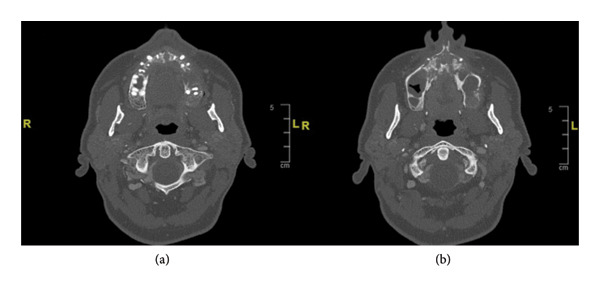
Initial imaging showing bone necrosis in the left tuberosity (a) and the upper part of the maxilla, involving the left maxillary sinus (b).

Advanced imaging demonstrated extensive maxillary necrosis spanning from tooth 15 to the left tuberosity, along with a small collection of fluid in the floor of the right nasal fossa (Figure 1).

**FIGURE 2:**
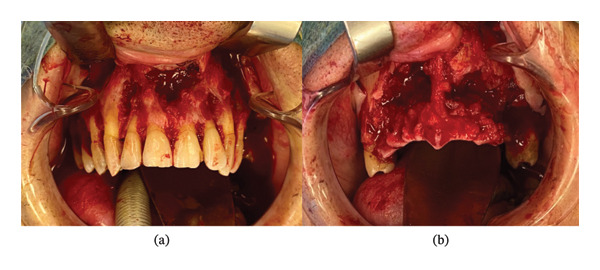
Intraoperative photographs showing the aspect of maxilla before (a) and after (b) limited debridement.

**FIGURE 3:**
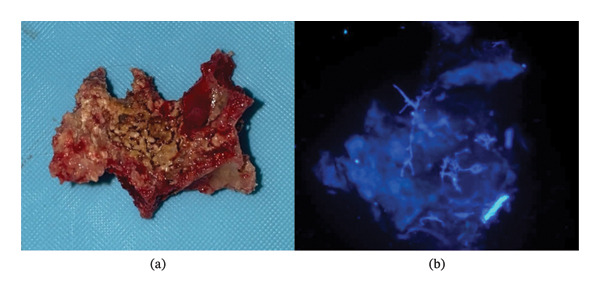
Intraoperative image showing necrotic bone with black fungal filaments (a) and immunofluorescence image showing mycelial filaments (b).

Laboratory results were unremarkable, with normal blood glucose (4.5 mmol/L), estimated glomerular filtration rate (eGFR; 90 mL/min), white blood cell (WBC) count (6.9 G/L), and C‐reactive protein (CRP) level (< 0.2 mg/L). Considering the patient’s history of testicular cancer and the post‐COVID‐19 context, potential diagnoses included metastatic spread of testicular cancer and post‐COVID‐19 fungal osteonecrosis, such as mucormycosis. A maxillary biopsy was performed under general anesthesia, involving minimal debridement of necrotic tissue and extraction of the mobile maxillary alveolar‐dental block from teeth 15 to 24, conserving teeth 16, 17, and 26 and the left tuberosity, which did not exhibit any mobility (Figure 2). No debridement was performed in the nasal or sinus floor. Multiple bone samples, including two necrotic 2‐cm blocks, were submitted for pathological analysis (Figure 3).

Histopathology (Figure 3), direct examination, and Mucorales polymerase chain reaction (PCR) confirmed mucormycosis. Immunological testing, human immunodeficiency virus (HIV) serology, and diabetes evaluations revealed no abnormalities. A positron emission tomography (PET) scan ruled out systemic involvement or secondary lesions but showed active disease in the anterior maxilla, the bony palate, and the maxillary sinus floor.

### 2.5. Intervention

Upon suspicion of mucormycosis, the patient received AmBisome combined with Tazocillin–clindamycin postoperatively. However, renal impairment (creatinine 113 µmol/L) required a switch to oral isavuconazole (200 mg every 8 h for 48 h, followed by 200 mg/day, later increased to 300 mg/day due to a residual isavuconazole level of 1.8 mg/L). A multidisciplinary discussion recommended extensive surgery, consistent with treatment guidelines, but the patient, a dental technician, initially refused extensive debridement due to the destructive nature of the procedure, potentially complicating dental rehabilitation. Long‐term antifungal therapy was maintained with close imaging and biological monitoring. Antibiotics were discontinued 6 weeks after the intervention.

### 2.6. Follow‐Up and Outcomes

**FIGURE 4:**
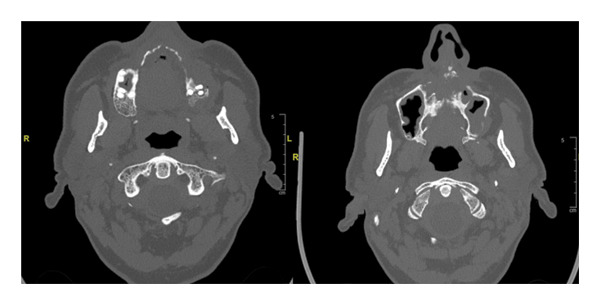
CT scan at 2 months postoperatively showing no disease progression.

Initial imaging (CBCT on Day 7 and MRI on Day 15, followed by alternating monthly MRI and CT scans) demonstrated stability without recurrence (Figure 4).

Three months after initial debridement, a sequestrectomy and extraction of tooth 27 were performed due to mobility, and isavuconazole was replaced with posaconazole due to abnormal liver function tests. Seven months postprocedure, an oroantral fistula was closed under local anesthesia using the Bichat flap, leading to satisfactory healing, and the patient was able to undergo rehabilitation with a removable prosthesis.

**FIGURE 5:**
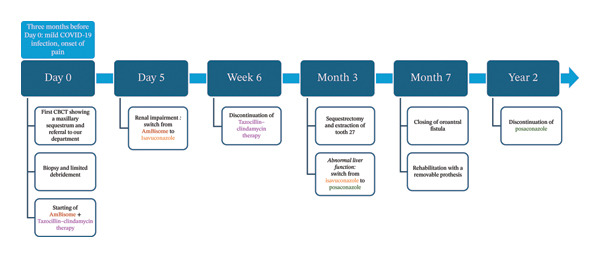
Timeline of medical care.

After 2 years of posaconazole therapy and biannual imaging showing no recurrence, antifungal treatment was discontinued (Figure 5).

### 2.7. Patient Perspective

As a dental technician, I was particularly concerned about the functional consequences of extensive surgery, which could have compromised any future dental rehabilitation. Although the initial phase of treatment was challenging, especially with the antifungal therapy complications, adjustments to the medication made it more bearable. I am satisfied with the conservative approach that was selected as I was able to proceed with rehabilitation using a removable dental prosthesis, which restored my ability to eat normally. Overall, despite the initial difficulties, the treatment and its outcomes were positive from my perspective.

## 3. Discussion

The present case of an immunocompetent patient raises several clinically relevant points, especially when compared with features of CAM. First, in systematic reviews of CAM, uncontrolled diabetes and steroid therapy or new‐onset hyperglycemia as a COVID‐19 complication were present in the majority of cases, and rhino‐orbital disease predominated, with mortality approaching 34% [16]. CAM often represents a “bridge” between classical immunocompromised hosts and patients with fewer known risk factors, but still with at least one modifiable predisposition. Our patient showed none of these conditions.

Second, while the European Confederation of Medical Mycology/Mycoses Study Group Education and Research Consortium global guidelines (2019) provide clear diagnostic and therapeutic pathways for patients with known risk factors, they lack guidance for those without such risk factors [17]. Current treatment guidelines for mucormycosis recommend extensive debridement for all patients. In contrast to most CAM cases that present with aggressive disease and require radical surgery, our patient’s favorable outcome with limited debridement suggests that immunocompetent hosts may be amenable to less aggressive surgical approaches, particularly when diagnosed early. In CAM cohorts, combined surgical and antifungal therapy is strongly associated with better survival, especially in rhino‐orbital mucormycosis without central nervous system involvement [12]. However, these data are largely derived from patients with comorbidities; the question of whether less extensive surgery suffices in immunocompetent patients is largely unexplored and merits prospective evaluation. A study involving patients who did not undergo surgical treatment revealed similar outcomes, although most of the patients did not exhibit bone involvement [18].

Third, the optimal medical therapy remains unclear. The recommended first‐line treatment is liposomal amphotericin B at a dose of 5–10 mg/kg, regardless of the extent of involvement. However, this therapy is associated with significant renal side effects, particularly because these patients often have other risk factors for renal insufficiency, such as diabetes. Isavuconazole represents an effective alternative, with hepatic elimination that spares renal function. It is recommended with moderate strength for the initial treatment of mucormycosis, along with posaconazole, or for salvage treatment in cases of inefficacy or renal intolerance to liposomal amphotericin B. Recent real‐world data support favorable safety and efficacy of isavuconazole, even when used as primary therapy, especially in patients at risk for amphotericin‐induced nephrotoxicity [19]. In CAM settings, azole agents have been used in salvage or combination regimens, yet no consensus exists on the optimal sequencing or combinations. These may be considered as the first‐line option, particularly in patients at risk of renal insufficiency.

The optimal treatment duration is also uncertain. Guidelines suggest continuing until reversal of immunosuppression and complete imaging resolution, with intravenous therapy until disease stabilization. In CAM cases, several published reports have continued therapy for weeks to months. However, some studies have demonstrated no recurrence after a short 15‐day treatment duration, even in patients with orbital floor invasion [18]. In this case, prolonged antifungal therapy was used without extensive surgical debridement. However, radiological and clinical normalization, as recommended by European guidelines, was achieved much earlier. The absence of recurrences indicates that, in immunocompetent patients with limited disease and good response, shorter therapy durations might suffice. These observations warrant further investigation into the minimum effective treatment duration.

Finally, comparing our immunocompetent case to CAM, it is noteworthy that in typical CAM cases, the pathophysiology often involves multiple synergistic conditions, including viral‐induced endothelial injury, immune dysregulation, lymphopenia, hyperglycemia, and corticosteroid exposure. These conditions promote fungal angioinvasion and proliferation. COVID‐19, even in mild forms, can transiently alter endothelial function, disrupt iron homeostasis, and modulate innate immunity, creating a permissive microenvironment for Mucorales invasion [13]. These atypical conditions may explain why some patients without chronic immunosuppression or traditional risk factors develop invasive disease.

In our case, the patient had no comorbidities other than a recent mild COVID‐19 infection. Mucormycosis developed despite the absence of severe viral illness or high‐dose corticosteroid therapy, suggesting that even subtle, transient immune or endothelial perturbations post‐COVID‐19 can lead to fungal proliferation.

In contrast to classical CAM, where multiple hits often drive rapid progression and extensive tissue involvement, our patient exhibited slower disease progression, limited local invasion, and preserved systemic immunity. This may explain the favorable outcome with relatively conservative surgical management and standard antifungal therapy. Nevertheless, this case highlights that even mild COVID‐19 can act as a contributing factor for mucormycosis in otherwise immunocompetent individuals, reinforcing the need for clinical vigilance and early diagnostic workup in patients presenting with compatible symptoms, regardless of COVID‐19 severity.

## 4. Conclusion

This case of upper jaw mucormycosis in an immunocompetent patient following a mild COVID‐19 infection highlights the potential for conservative treatment strategies, including minimal debridement and short‐term antifungal therapy. The favorable outcome with a conservative strategy challenges current guidelines that emphasize aggressive surgical intervention and underscores the need for further investigation into treatment duration and first‐line antifungal options for this rare presentation. This approach seems particularly interesting in cases of rhino‐orbital mucormycosis, where the functional consequences in terms of vision or diet can have a major impact on patients’ quality of life.

## Funding

This work received no funding.

## Disclosure

This study was performed as part of the employment for Hospices Civils de Lyon.

## Ethics Statement

In accordance with the ethical guidelines for case report publication, written informed consent for publication was obtained from the patient.

## Conflicts of Interest

The authors declare no conflicts of interest.

## Data Availability

Data sharing is not applicable to this article as no datasets were generated or analyzed during the current study.
